# Perioperative Heparin Bridging in Patients With Mechanical Aortic Valves Undergoing Elective Surgery: The AMBER Study

**DOI:** 10.1016/j.jacadv.2025.102578

**Published:** 2026-01-23

**Authors:** Alejandro Godoy, Tegvir S. Grewal, Vinai Bhagirath, Alfonso Tafur, Amelia McNiven Fontani, Ana I. Casanegra, Atefeh Ghorbanzadeh, Damon E. Houghton, Jameel Abdulrehman, Jean-Philippe Galanaud, Karen Sidhom, Luigi Del Sordo, Mouza Alnuaimi, Paul R. Daniels, Stephanie Carlin, Yama Sadozai, Alex Spyropoulos, James Douketis

**Affiliations:** a Population Health Research Institute, Hamilton, Ontario, Canada; b Department of Thrombosis, Hamilton Health Sciences Corporation, Hamilton, Ontario, Canada; c Department of Medicine, McMaster University, Hamilton, Ontario, Canada; d Vascular Medicine and Thrombosis Service, Hospital Privado Universitario de Córdoba, Córdoba, Argentina; e School of Medicine, Queen’s University, Kingston, Ontario, Canada; f Department of Medicine, Endeavor Health, Evansville, Illinois, USA; g Gonda Vascular Center, Mayo Clinic, Rochester, Minnesota, USA; h University Health Network, University of Toronto, Toronto, Ontario, Canada; i Department of Medicine, Sunnybrook Health Sciences Centre, Toronto, Ontario, Canada; j Department of Medicine, Northwell Health at Lenox Hill Hospital, New York, New York, USA; k Department of Medicine, St. Joseph’s Healthcare Hamilton, Hamilton, Ontario, Canada

**Keywords:** bleeding, heparin bridging, low-molecular-weight heparin, mechanical aortic valve, perioperative management, thromboembolism

## Abstract

**Background:**

The benefit–risk balance of perioperative low-molecular-weight heparin (LMWH) bridging in patients with bileaflet mechanical aortic valves undergoing elective procedures is uncertain. While intended to prevent thromboembolism during warfarin interruption, LMWH may increase bleeding risk without a proven efficacy benefit.

**Objectives:**

This study aimed to evaluate the rates of thromboembolism and bleeding associated with different peri-operative LMWH bridging strategies compared to no bridging in this patient population.

**Methods:**

In a multicenter retrospective cohort study, 553 patients requiring warfarin interruption were classified into 3 main analysis groups: preoperative and postoperative LMWH (n = 232), preoperative-only LMWH (n = 81), postoperative-only LMWH (n = 33), and no bridging (n = 207). The primary efficacy outcome was arterial thromboembolism within 30 days. Primary and secondary safety outcomes were major bleeding and clinically relevant non-major bleeding. Outcomes were analyzed with multivariable logistic regression and propensity-matched comparisons.

**Results:**

Thromboembolic events were rare (<0.5%) and similar across all strategies. Compared to no bridging, full bridging was associated with significantly higher rates of major bleeding (3.0% vs 0%; *P* = 0.018) and any clinically relevant bleeding (9.5% vs 2.0%; *P* < 0.01). In multivariable models, full bridging predicted bleeding, whereas preoperative-only bridging did not. In a propensity-matched analysis, full bridging remained associated with a higher risk of any clinically relevant bleeding (10.6% vs 2.4%; *P* < 0.01).

**Conclusions:**

Among patients with bileaflet mechanical aortic valves undergoing elective procedures, 30-day thromboembolic events were rare. Full perioperative LMWH bridging was associated with increased bleeding. While this study provides valuable real-world data, prospective clinical trials are necessary to confirm and expand upon these findings.

In patients with a mechanical heart valve (MHV) who are receiving a vitamin K antagonist such as warfarin, approximately 1 in 5 will require an elective surgery or invasive procedure every year.^1^ Peri-operative heparin bridging is defined as the administration of a short-acting parenteral anticoagulant, typically a low-molecular-weight heparin (LMWH), during the perioperative period when warfarin is temporarily interrupted and the international normalized ratio falls below therapeutic range. LMWH bridging is generally reserved for selected patients considered to be at high risk of thromboembolism and is typically administered for 3 days before and 3 to 5 days after an elective surgery/procedure.^2^^,^^3^

In warfarin-treated patients with atrial fibrillation (AF) undergoing elective surgeries or procedures, LMWH bridging does not reduce the risk of thromboembolism but is associated with a 3-fold increase in the risk of major perioperative bleeding.^4^ Consequently, clinical practice guidelines generally recommend against bridging in most patients with AF receiving warfarin during perioperative anticoagulant interruption.^3^ However, in patients anticoagulated for an MHV, the benefit of perioperative bridging remains uncertain. Observational studies demonstrate variability in bridging practices and imprecision in the rates of thromboembolism and bleeding.^3^^,^^5^^,^^6^

Most of the evidence supporting perioperative LMWH bridging in patients with an MHV comes from cohort studies without a comparator group.^7^, ^8^, ^9^ The only randomized trial comparing bridging with no bridging in this population was underpowered to detect differences in outcomes, and all patients received preoperative LMWH bridging.^10^ Not surprisingly, practice guidelines offer conditional or weak recommendations for periprocedural bridging around an elective surgery/procedure: bridging is typically suggested for patients with a mitral MHV, whereas recommendations vary for patients with an aortic MHV, which is the most common type of MHV.^1^^,^^2^^,^^11^

Against this background, the aim of this study was to determine the rates of perioperative thromboembolism and bleeding in patients with a bileaflet aortic MHV undergoing elective surgeries or procedures, comparing thromboembolic and bleeding outcomes across different perioperative bridging strategies.

## Methods

### Study design

This was a multicenter, retrospective cohort study assessing perioperative clinical outcomes (thromboembolism and bleeding) in patients with a bileaflet aortic MHV, requiring warfarin interruption for an elective surgery or procedure. The study was conducted at 5 centers in the United States and Canada (Hamilton General Hospital, Hamilton Ontario), St. Joseph’s Healthcare, Hamilton, Ontario, Mayo Clinic, Rochester, Minnesota, NorthShore Health System, Evansville, Illinois, and Sunnybrook Health Sciences Centre, Toronto, Ontario) and was approved by the Institutional Review Boards at each center.

### Patients

Consecutive eligible patients were identified from the electronic medical records of participating centers over a 3-year period, from July 1, 2020 until June 30, 2023. Specifically, patients were identified by searching records from one or more of peri-operative assessment clinics, anticoagulation management clinics, and perioperative anticoagulation/bridging clinics.

Patients were included if they satisfied all of the following criteria: 1) age ≥18 years; 2) presence of bileaflet aortic MHV; 3) underwent an elective surgery/procedure that required warfarin interruption; and 4) had documentation of follow-up for at least 30 days after the surgery/procedure. Patients were excluded if they had one or more of the following criteria: 1) presence of a mechanical mitral valve; 2) underwent multiple procedures during the 30-day postoperative period; and 3) did not have at least one encounter during the 30-day follow-up.

### Definition and classification of perioperative heparin bridging

Heparin bridging was defined as the use of a once- or twice-daily LMWH regimen, comprising either enoxaparin 1 mg/kg twice daily, enoxaparin 1.5 mg/kg once daily, dalteparin 100 international units (IU)/kg twice daily or 200 IU/kg once daily, or tinzaparin 175 IU/kg once daily.

Patients were initially categorized into 4 peri-operative LMWH bridging strategy groups: 1) preoperative and postoperative bridging; 2) preoperative bridging only; 3) postoperative bridging only; and 4) no bridging. For the primary analyses, we compared bridging (preoperative and postoperative or preoperative only) vs no bridging. Patients who received postoperative bridging only were excluded from comparative analyses because they represented only 6% of the cohort and this strategy is not routinely used in clinical practice. Surgery/procedure bleeding risk was classified according to prespecified, empirically derived, criteria outlined in the International Society on Thrombosis and Haemostasis Scientific and Standardization Committee guidance document on periprocedural anticoagulant management and was categorized as high-bleed-risk, low-/moderate-bleed-risk, or minimal-bleed-risk.^12^

### Study outcomes

The primary efficacy outcome was thromboembolism requiring hospitalization or further treatment, including both arterial and venous events. Arterial thromboembolism comprised ischemic stroke and systemic embolism (eg, acute limb ischemia, bowel ischemia, splanchnic infarction, or kidney infarction). Venous thromboembolism included deep vein thrombosis and pulmonary embolism. Valve thrombosis was defined as any imaging-confirmed thrombus on a mechanical valve prosthesis associated with relevant clinical signs or symptoms. The primary safety outcome was major bleeding (MB) or clinically relevant non-major bleeding (CRNMB), as defined by the International Society on Thrombosis and Haemostasis.^13^ A secondary safety outcome was any clinically relevant bleeding (CRB), defined as the composite of MB and CRNMB. All outcomes occurring within the 30-day postoperative period were included in the study and were adjudicated by the principal investigator at each site.

### Statistical analysis

Descriptive and comparative statistical analyses were conducted to evaluate differences between bridging strategies. Categorical variables were compared using the chi-square or Fisher exact test, and ORs were estimated. Normally distributed continuous variables were compared using the Student’s *t*-test, while non-normally distributed variables were analyzed using nonparametric tests (Mann-Whitney *U*). Logistic regression analyses were used to evaluate the effect of the bridging strategy and other covariates on bleeding outcomes. Due to the low number of thrombotic events, recreation analysis was not performed for efficacy outcomes. A multivariate logistic regression using a stepwise (forward) selection method was used to identify independent predictors of bleeding.

To address potential confounding in this observational study, analyses were performed using propensity score matching between groups with balanced baseline characteristics. First, the effect of full (preoperative and postoperative) LMWH bridging was compared to a nonbridging management. For this analysis, a propensity score was estimated for each patient based on covariates previously associated with the outcomes (*P* < 0.10). These covariates included hypertension, chronic kidney disease (CKD), history of AF, time since valve replacement, patient age, CHADS_2_ score, surgery/procedure bleeding risk, doubling of the initial postoperative warfarin dose, and whether the procedure was gastroenterological. Matching was done 1:1 using nearest-neighbor matching with a caliper of 0.2 SDs of the logit of the propensity score. Subsequently, a second propensity score analysis was done to compare the effect of LMWH bridging given only preoperatively vs no bridging. This analysis applied the same set of covariates and the identical matching methodology.

Patients who received postoperative bridging alone were not included in regression or propensity score analyses, as this management strategy is rarely used in clinical practice and was not considered clinically relevant for comparative evaluation.

## Results

### Patients

We assessed 553 patients with a bileaflet aortic MHV who required perioperative warfarin interruption. Baseline clinical characteristics are shown in Table 1. Perioperative LMWH bridging management was distributed as follows: a total of 232 patients (42.0%) received both preoperative and postoperative LMWH bridging; 81 (14.6%) received preoperative LMWH bridging only; 33 (6%) received postoperative LMWH bridging only, and 207 (37.4%) received no bridging. Further details on the distribution of procedures and their bleeding risk, anesthesia use, perioperative anticoagulation and antiplatelet strategies, and CHADS_2_ scores are available in Supplemental Tables 1 to 3.

**Table 1:** Baseline Patient Characteristics

				*P* Value	
	Preoperative and Postoperative Bridging (n = 232)	No Bridging (n = 207)	Preoperative Bridging (n = 81)	Preoperative/Postoperative Bridging vs No Bridging	Preoperative/Postoperative Bridging vs Preoperative Bridging
Female	49 (21.1%)	52 (25.1%)	23 (28.4%)	0.38	0.21
Age (y, IQR)	65 (60-73)	64 (57,6-72)	66 (58-74)	0.70	0.59
Diabetes	62 (26.7%)	58 (28.0%)	22 (27.2%)	0.84	0.92
Hypertension	172 (74.1%)	132 (63.8%)	64 (79.0%)	0.02	0.37
Chronic kidney disease	43 (18.5%)	46 (22.2%)	7 (8.64%)	0.40	0.03
Congestive heart failure	56 (24.1%)	50 (24,15%)	14 (17.3%)	0.024	0.02
Coronary artery disease	100 (43.1%)	64 (30.92%)	28 (34.6%)	<0.001	0.20
Peripheral artery disease	23 (9.91%)	17 (8,21%)	5 (6.17%)	0.029	0.36
History of revascularization	70 (30.2%)	23 (11.1%)	28 (34.6%)	<0.001	0.46
History of stroke or TIA	47 (20.3%)	10 (4,83%)	11 (33,3%)	<0.001	0.55
History of VTE	19 (8.19%)	9 (4,35%)	3 (3.70%)	0.031	0.16
History of atrial fibrillation	100 (43.1%)	69 (33,33)	26 (32.1%)	0.118	0.09
CHADS_2_ (median, IQR)	2 (1-3)	1 (1-2)	2 (1-2)	0.48	0.52
High-bleed-risk surgery	61 (26.3%)	41 (19.8%)	19 (23.5%)	0.14	0.62
Moderate-bleed-risk surgery	123 (46.9%)	122 (58.9%)	57 (70.4%)	0.25	<0.001
Minimal-bleed-risk surgery	19 (8.19%)	22 (10.6%)	21 (25.9%)	0.48	<0.001
Perioperative antiplatelet interruption	22 (9.48%)	21 (18,92%)	10 (50%)	0.02	<0.001
Perioperative antiplatelet continuation	92 (39.7%)	111 (53,62)	21 (26,28)	<0.001	<0.001

TIA = transient ischemic attack; VTE = venous thromboembolism.

Values given as number (%) unless clarified.

### Direct comparisons of rates of thromboembolism, bleeding, and mortality

The 30-day incidence of postoperative clinical outcomes is shown in Table 2. Arterial thromboembolic events were rare and occurred at similar rates in the preoperative and postoperative bridging group (n = 1, 0.43%) and the no bridging group (n = 1, 0.48%). Venous thromboembolism occurred in only one patient (0.43%) in the no bridging group. No valve thrombotic events were reported.

**Table 2:** Bleeding and Thromboembolic Outcomes by Perioperative Bridging Strategy

				*P* Value	
	Preoperative and Postoperative Bridging (n = 232)	No Bridging (n = 207)	Preoperative Bridging (n = 81)	Preoperative and Postoperative Bridging vs No Bridging	Preoperative and Postoperative Bridging vs Preoperative Bridging
Bleeding					
Major bleeding	7 (3.02%)	0	1 (1.23%)	**0.018**	0.68
Clinically relevant non-major bleeding	15 (6.47%)	5 (2.04%)	2 (2.47%)	**0.029**	0.27
Any bleeding	22 (9.48%)	5 (2.04%)	3 (3.70%)	**0.0009**	0.17
Thromboembolic					
Stroke/systemic embolism	1 (0.43%)	1 (0.48%)	0	1.00	1.00
Myocardial infarction	0	0	0	-	-
Valve thrombosis	0	0	0	-	-
Venous thromboembolism	0	1 (0.41%)	0	1.00	-
All-cause mortality	1 (0.43%)	1 (0.41%)	0	1.00	1.00

Values given as number (%).

Patients who received both preoperative and postoperative bridging experienced significantly higher rates of bleeding compared to those who received no bridging. This was consistent for MB (3.0% vs 0%; *P* = 0.018), CRNMB (6.5% vs 2.0%; *P* = 0.029), and any CRB (9.5% vs 2.0%; *P* < 0.01).

There were 2 deaths during follow-up: one in the preoperative and postoperative bridging group (0.43%) and one in the no bridging group (0.41%). Neither death was related to a thromboembolic or bleeding event.

### Univariate and multivariate regression analysis

#### Major bleeding

In the univariate regression analysis for MB, the association with LMWH bridging was assessed by comparing any bridging strategy (preoperative and postoperative or preoperative only) vs no bridging. Patients who received postoperative bridging only were excluded from this analysis due to their small number (6% of the cohort). The incidence of MB was 3.0% in the preoperative and postoperative bridging group, 1.2% in the preoperative-only bridging group, and 0% in the no bridging group. Congestive heart failure (*P* = 0.039) and colonoscopy (*P* = 0.021) were associated with a lower likelihood of MB, although the extreme negative coefficients suggest possible data sparsity in these subgroups.

In the multivariate regression analysis using a stepwise selection method, 2 variables emerged as independent predictors of MB: any LMWH bridging (preoperative and postoperative or preoperative only; *P* = 0.030), which increased bleeding risk and doubling the first postoperative warfarin dose (*P* = 0.027), which was associated with reduced risk. The lowest predicted probability of MB (<0.2%) was observed in patients undergoing minimal surgical bleeding risk procedures, who received no LMWH bridging, and had the first postoperative dose of warfarin doubled. The predicted probability of MB increased with age, reaching its highest level (50.3%) among older patients undergoing a high surgical bleeding–risk procedure, who received postoperative-only LMWH bridging and did not have their warfarin dose doubled.

#### Clinically relevant non-major bleeding

Univariate logistic regression did not identify a statistically significant association between CRNMB and bridging strategy (*P* = 0.14).

#### Any clinically relevant bleeding

In the univariate analysis, the LMWH bridging strategy was significantly associated with an increased risk of any CRB (*P* = 0.004). The estimated probabilities of bleeding were 9.5% in the preoperative and postoperative bridging group, 3.7% in the preoperative-only bridging group, and 2.4% in the no bridging group.

In the stepwise multivariate logistic regression model for predicting any CRB, 2 variables were retained as independent predictors: surgical bleeding risk category (*P* = 0.03) and LMWH bridging strategy (*P* = 0.04). These variables entered the model in order of importance. The model estimated the lowest probability of bleeding (1.45%) for patients undergoing minimal-risk procedures without bridging. In contrast, the highest probability for bleeding of 17.7% was observed in patients undergoing high-bleed-risk who received both preoperative and postoperative bridging.

In the univariate regression analysis for any CRB, additional factors significantly associated with increased risk included hypertension (OR: 3.22; *P* = 0.009), CKD (OR: 2.47; *P* = 0.044), age (OR: 1.05 per year; *P* = 0.011), and CHADS_2_ score (OR: 1.39 per point; *P* = 0.012). Surgical bleeding risk category was also predictive of CRB (*P* = 0.004), with estimated bleeding rates of 11.5% for high-risk, 3.6% for low-/moderate-risk, and 3.2% for minimal-risk surgeries/procedures. Protective factors for CRB included doubling the first postoperative warfarin dose (OR: 0.29; *P* = 0.013) and undergoing a gastroenterological procedure (OR: 0.25; *P* = 0.002) (Supplemental Table 5).

### Propensity score matching sensitivity analyses

To mitigate the effects of potential confounding from baseline patient characteristics, a 1:1 propensity score-matched analysis was performed. The first analysis compared patients who received both preoperative and postoperative bridging with those who received no bridging. From 439 eligible patients (232 in the preoperative and postoperative bridging group, and 207 in the no bridging group), a matched cohort of 414 patients (207 in each group) was generated. Baseline characteristics were well balanced after matching. In this cohort, patients who received prebridging and postbridging had significantly higher rates of CRNMB (7.3% vs 2.4%; *P* = 0.027), and any CRB (10.6% vs 2.4%; *P* < 0.01). There was no significant difference in MB (*P* = 0.85). A second propensity score-matched analysis compared patients receiving preprocedure bridging only with those receiving no bridging. No statistically significant differences were found in the rates of CRNMB, and any CRB bleeding was not analyzed due to the low number of events (Central Illustration).

**Central Illustration:**
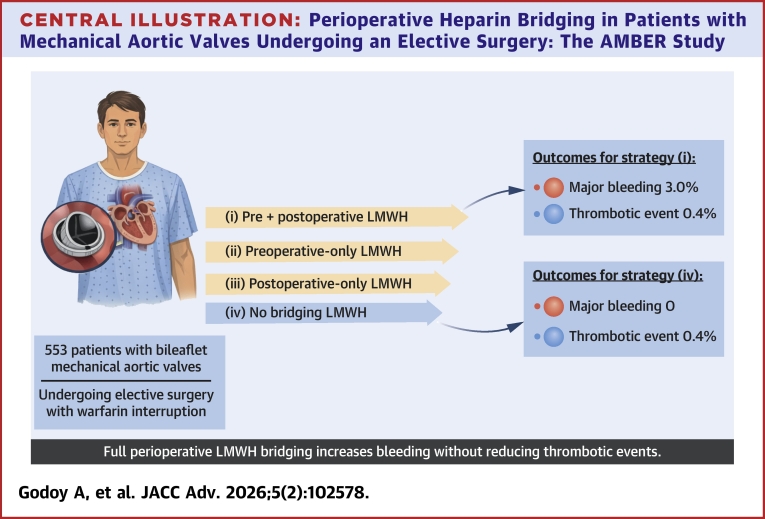
Perioperative Heparin Bridging in Patients with Mechanical Aortic Valves Undergoing an Elective Surgery: The AMBER Study The AMBER (Aortic Mechanical valve Bridging Evaluation in suRgery) Study evaluated 553 patients with bileaflet mechanical aortic valves who required peri-operative warfarin interruption for elective surgery. Strategies were compared: preoperative and postoperative LMWH, preoperative-only LMWH, and no bridging. Thromboembolic events were rare and comparable across all strategies, while preoperative and postoperative LMWH bridging was associated with significantly higher bleeding rates. LMWH = low-molecular-weight heparin.

## Discussion

In this study evaluating perioperative anticoagulation strategies in patients with mechanical bileaflet aortic valves undergoing elective surgery or procedures, thromboembolic events—including stroke, systemic embolism, venous thromboembolism, and valve thrombosis—as well as all-cause mortality were infrequent across all groups and did not appear to differ based on use or nonuse of LMWH bridging. In contrast, patients managed with both preoperative and postoperative LMWH bridging experienced significantly higher rates of MB, CRNMB, and overall CRB compared to those managed without bridging.

In the multivariable regression analysis, preoperative and postoperative LMWH bridging was independently associated with a significantly increased risk of both MB and any CRB. Although this strategy was not significantly associated with CRNMB in the multivariate model, the propensity score matching analysis confirmed that both any CRB and CRNMB remained significantly higher in the preoperative and postoperative bridging group compared to the no bridging group. Patients who received preoperative bridging only did not show a significant increase in bleeding risk, and postoperative bridging only was infrequent and not included in the main analyses.

It is important to distinguish the clinical implications of MB and CRNMB when interpreting the risk–benefit balance of perioperative bridging. MB carries substantial morbidity, including potential need for transfusion, surgical intervention, and mortality risk. CRNMB, while less severe, remains clinically meaningful as these events often require medical intervention, may prolong hospitalization, delay anticoagulation resumption, and adversely affect patient recovery and quality of life. Both types of bleeding contribute to the overall safety profile of perioperative management strategies and must be considered when weighing the benefits of thromboprophylaxis against bleeding risk in patients with mechanical aortic valves.

These findings align with results from randomized trials comparing perioperative anticoagulation strategies with and without LMWH bridging.^4^^,^^5^ Specifically, our observed rates of MB, that is 3.0% in the preoperative and postoperative bridging group and 0% in the no bridging group, are consistent with findings from the BRIDGE (Bridging Anticoagulation in Patients Who Require Temporary Interruption of Warfarin Therapy for an Elective Invasive Procedure or Surgery) trial, which reported MB rates of 3.2% and 1.3%, respectively, in patients with AF managed with or without bridging.^4^ Similarly, our observed 1.2% rate of MB in patients receiving preoperative bridging alone is comparable to the 2.0% rate reported in the PERIOP-2 (Postoperative Low-Molecular-Weight Heparin Bridging Treatment for Patients at High Risk of Arterial Thromboembolism) trial, which included patients with AF or an MHV, all of whom received preprocedural LMWH and were randomized to postprocedural LMWH vs placebo. In PERIOP-2, the MB rate was 1.96% in the postbridging group and 0.67% in the no postbridging group. CRNMB was also more frequent with postprocedural bridging (6.1% vs 3.9%), findings that are consistent with our observation of increased bleeding risk when LMWH is continued after the procedure.^5^

Our finding of low (∼0.4%) rates of stroke or systemic embolism, regardless of bridging strategy, is also consistent with findings from the MHV subgroup of the PERIOP-2 trial, where such events occurred at similarly low rates (∼0.5%), irrespective of bridging strategy. These results suggest that the intrinsic arterial thromboembolic risk in patients with bileaflet aortic valves undergoing perioperative anticoagulants interruption is low and comparable to the 0.3% to 0.4% risk of stroke/systemic embolism rates observed in patients with AF managed without LMWH bridging.^4^^,^^14^ This aligns with emerging evidence from the LIMIT (Lowering the International Normalized Ratio Target in Patients With Mechanical Aortic Valves) trial, which is evaluating whether lower international normalized ratio targets in patients with bileaflet aortic MHVs may be sufficient to maintain thromboembolic protection while potentially reducing bleeding risk.

### Study Limitations

This study has several potential limitations. First, its retrospective observational design and nonrandom allocation of patients to bridging or no bridging strategies introduce inherent bias. Although we used propensity score matching and multivariable adjustment to mitigate confounding, these methods cannot fully eliminate bias and causal inferences should be made cautiously. The decision to use LMWH bridging was not randomized and likely reflected clinician judgment based on perceived thrombotic risk, potentially influenced by unmeasured factors such as valve characteristics, recent thromboembolic events, or coagulation abnormalities. Additionally, the absence of individual CHA_2_DS_2_-VASc scores limits our ability to precisely stratify thromboembolic risk and may have affected the comparability of risk profiles between groups. Importantly, several risk factors for thromboembolism—such as advanced age, AF, hypertension, and CKD—are also associated with bleeding risk; therefore, patients who received bridging may paradoxically have been at higher baseline risk for both thromboembolism and bleeding, limiting comparability with nonbridged patients. Moreover, if clinicians tended to withhold bridging only in patients perceived to be at low thromboembolic risk, and if bridging were highly effective, our results could theoretically underestimate its efficacy. However, the overall low incidence of thromboembolic events across all groups suggests that such bias is unlikely to have substantially influenced the main findings. Second, we did not apply formal adjustments for multiple comparisons. While this may increase the risk of type I error, our primary comparisons were prespecified and limited, and *P* values should be interpreted as descriptive rather than confirmatory in this observational study. Third, the exceedingly low incidence of thromboembolic events in our study substantially limits our statistical power to detect differences in efficacy outcomes between bridging strategies. This precludes definitive conclusions about the comparative effectiveness of different approaches in preventing thromboembolism. While the consistently low event rates across all groups—including those receiving no bridging—suggest that thrombotic risk may be lower than historically reported in this population, we cannot exclude the possibility that larger studies might reveal clinically important differences. Therefore, our findings regarding thromboembolic outcomes should be interpreted with appropriate caution, and the study's primary contribution lies in demonstrating the significant bleeding risk associated with full peri-operative bridging rather than establishing noninferiority or equivalence for thromboprophylaxis. Fourth, clinical outcomes were adjudicated locally and without blinding to treatment status, introducing the possibility of ascertainment bias. It is possible that we may have missed some minor bleeding or thrombotic events; however, it is unlikely that important or clinically significant events were overlooked. Moreover, the consistency of our outcome rates with those reported in trials with blinded adjudication supports the accuracy of our classifications.^4^^,^^14^ Finally, the generalizability of our findings is limited to patients with bileaflet mechanical aortic valves undergoing elective procedures with stable therapeutic anticoagulation. These results may not apply to patients with older-generation monoleaflet aortic valves, newer-generation On-X valves, mechanical mitral valves nor to individuals at markedly elevated thromboembolic risk, such as those with prior perioperative thromboembolism or recent stroke or systemic embolism.

There are strengths of this study that support the accuracy and reliability of the findings. First, the inclusion of multiple centers from Canada and the United States enhances generalizability and reflects real-world practices across diverse clinical settings and health care systems. Second, focusing exclusively on patients with a bileaflet aortic MHV—the most commonly used prosthetic valve—further supports the relevance of the findings, as patients with other valve types may have different thromboembolic risk.^15^ Third, the inclusion of sites with specialized thrombosis and/or anticoagulation clinics likely ensured high-quality clinical care and reliable event capture. Fourth, the relatively large sample size strengthens the precision of our estimates and supports the robustness of our results. Finally, clinical outcomes were adjudicated using standardized definitions and verified by thrombosis specialists, further supporting the validity of the findings.

## Conclusions

Among patients with mechanical bileaflet aortic valves requiring perioperative warfarin interruption, thromboembolic events remained low and similar across groups, regardless of bridging strategy. In contrast, management with preoperative and postoperative LMWH bridging was associated with a significantly higher risk of bleeding compared to no bridging. These findings raise important questions about the net clinical benefit of routine LMWH bridging in this population and suggest that a more selective, risk-stratified approach may be warranted. While this study provides valuable real-world data, prospective clinical trials are necessary to confirm and expand upon these findings.

## Funding support and author disclosures

Dr Bhagirath has received honoraria from 10.13039/100004326Bayer. Dr Tafur has received historical research support from Bristol Myers Squibb (BMS), BIOTAP, DOASENSE, Stago, Janssen, and Idorsia; in the past year, he has received research support from 10.13039/100020132Anthos Therapeutics and the 10.13039/100015438International Society on Thrombosis and Haemostasis (ISTH). He previously has served as a consultant to Janssen and Recovery Force and has served on the Board of Directors of VascuLearn, a 501(c) (3) nonprofit organization. Dr Carlin has received honoraria and/or advisory board fees from 10.13039/100004325AstraZeneca, 10.13039/100017530Fresenius Kabi, 10.13039/501100023331Leo Pharma, 10.13039/100004319Pfizer/10.13039/100002491BMS, and 10.13039/501100011725Servier. All other authors have reported that they have no relationships relevant to the contents of this paper to disclose.
