# Supplementary material for: Perioperative Heparin Bridging in Patients With Mechanical Aortic Valves Undergoing Elective Surgery: The AMBER Study
Source: JACC Adv. 2026 Jan 23;5(2):102578. doi: 10.1016/j.jacadv.2025.102578 (PMC12860947; doi:10.1016/j.jacadv.2025.102578)
Supplement: Supplemental Tables 1-5 [file mmc1.docx]

| Procedure Category |  | n (%) |
| --- | --- | --- |
| *****High Bleed-Risk Procedures***** |  | **170** |
| Cancer surgery | Solid tumor resection (lung, esophagus, gastric, colon, hepatobiliary, pancreatic) | 10 (5.88%) |
| Major orthopedic surgery | Shoulder replacement, total knee arthroplasty | 33 (19.4%) |
| Reconstructive & major thoracic surgery | Plastic or thoracic reconstruction | 16 (9.42%) |
| Major GI or urologic surgery | Bowel resection, anastomosis, colonic polyp resection, nephrectomy | 14 (8.24%) |
| Procedures in vascular organs | Liver, kidney, pancreas, spleen | 5 (2.94%) |
| Neurosurgery or spinal procedures | Intracranial or spinal surgery, epidural injections | 13 (7.65%) |
| Cardiac and vascular surgery | CABG, valve, or peripheral arterial surgery | 17 (10.0%) |
| Head & neck surgery | ENT cancer resections, extensive oral cavity procedures | 9 (5.29%) |
| Other high-risk procedures | Kidney biopsy, PEG, ERCP, partial breast mastectomy, tangential skin grafting | 8 (4.71%) |

| *****Low-to-Moderate Bleed-Risk Procedures***** |  | 315 |
| --- | --- | --- |
| Endoscopic procedures | GI endoscopy, colonoscopy, or both | 192 (61.0%) |
| Minimally invasive abdominal surgery | Laparoscopic cholecystectomy, appendectomy, hernia repair, dialysis catheter insertion | 28 (8.89%) |
| Thoracic/airway procedures | Bronchoscopy with biopsy | 6 (1.90%) |
| Cardiac catheterization | Coronary and peripheral angiography | 36 (11.4%) |
| Soft tissue & orthopedic minor surgery | Arthroscopy, hand/foot surgery, minor ortho | 28 (8.89%) |
| Biopsies | Cutaneous, lymph node, breast (ultrasound-guided) | 14 (4.44%) |
| Other low/moderate-risk procedures | Hysteroscopy, sternal wound debridement, joint injections, mitral valve repair (transcatheter), central lines, minor oncologic surgery | 11 (3.49%) |
| *****Minimal Bleed-Risk Procedures***** |  | \|  \| \| --- \|  \| **68** \| \| --- \| |
| Dermatologic | Excision of BCC/SCC, actinic keratoses, nevi | 14 (20.6%) |
| Ophthalmologic | Cataract surgery and similar | 16 (23.5%) |
| Dental | Cleanings, restorations, extractions, prosthetics | 19 (27.9%) |
| Device-related | Pacemaker/ICD insertion, port-a-cath | 14 (20.6%) |
| Other minimal-risk procedures | Dorsal split, carpal tunnel injection, temporal artery biopsy | 5 (7.35%) |

**Table S1.** Type of procedure & bleeding risk

| Use of Neuraxial Anesthesia | n (%) |
| --- | --- |
| Yes | 9 (4.81%) |
| No | 178 (95.2%) |

**Table S2.** Neuraxial Anesthesia Use

| Received LMWH pre procedure  Number of Days Before Procedure  1  2  3  4  Unknown | 307  272 (89.0%)  14 (4.56%)  9 (2.93%)  1 (3.23%)  8 (2.61%) |
| --- | --- |

**Table S3.** Date of last dosage of LMWH pre procedure.

| LMWH Dosages  Type of LMWH  Enoxaparin  Tinzaparin  Dalteparin | 290  240  30  20 |
| --- | --- |

**Table S4.** Type of LMWH used.

| Age/Sex | Procedure | VKA Dose | Bridging | LMWH | Antiplatelet | Event |
| --- | --- | --- | --- | --- | --- | --- |
| 58M | GI endoscopy w/ biopsy | 17.5 mg/week | No | N/A | ASA | Death (Vfib arrest), 13d post-op |
| 64M | Needle aspiration | 22.5 mg/week | Yes | Enoxaparin | None | Death (unknown cause) |
| 78M | Coronary angiography | 85 mg/week | No | N/A | None | Ischemic stroke, day of procedure |
| 74M | Cystectomy w/ ileal conduit | 8 mg/week | No | Prophylactic post-op | ASA | Stroke (day 25), MB (day 5) |
| 60M | PORT A CATH placement | 21 mg/week | Yes | Enoxaparin BID post-op | None | Systemic embolism (septic to brain) |
| 78M | GI endoscopy | 37.5 mg/week | Yes | Dalteparin | None | Major bleeding (day 12) |
| 72M | Abdominal hernia repair | 35 mg/week | Yes | Enoxaparin | None | Major bleeding |
| 75M | Orthopedic surgery | 35 mg/week | Yes | Enoxaparin + UFH | None | Major bleeding |
| 55M | CABG + AVR | 34.5 mg/week | Yes | Enoxaparin pre-op | ASA | MB day 2, INR 7.2 |
| 77M | TRUS biopsy | 42.5 mg/week | Yes | Enoxaparin | None | Major bleeding |
| 57M | Laparoscopic cholecystectomy | 70 mg/week | Yes | Dalteparin | ASA | Major bleeding (day 8) |
| 68M | Parotidectomy + neck dissection | 21 mg/week | Yes | Enoxaparin | ASA | Major bleeding (day 10) |
| 66M | Laparoscopic cholecystectomy | 45 mg/week | No | Prophylactic | None | Major bleeding (day 5) |
| 64M | GI endoscopy | 42.5 mg/week | Yes | Enoxaparin post-op | None | Major bleeding |
| 76M | Thyroidectomy + neck dissection | 37.5 mg/week | Yes | Enoxaparin | None | Major Bleeding, day 5 |
| 76M | TRUS biopsy | 42.5 mg/week | Yes | Enoxaparin | None | Major bleeding, day 7 |
| 54F | Colonoscopy + GI endoscopy w/ biopsy | 21 mg/week | Yes | Dalteparin | None | CRNMB, day 18 |
| 68F | Colonoscopy | 28 mg/week | Yes | Enoxaparin | ASA | CRNMB, day 10 |
| 81M | Cerebral Angiogram + MMA Embolization | 35 mg/week | No | None | ASA stopped | CRNMB, post-op |
| 55M | Renal transplant | 40 mg/week | No | Prophylactic | None | CRNMB, day 10 |
| 74M | Cystoscopy biopsy | 15 mg/week | No | None | ASA | CRNMB, day 13 |
| 73M | Lumbar decompression | 29 mg/week | No | Unknown | ASA | CRNMB, day 25 |
| 72F | PEG placement | 35 mg/week | Yes | Enoxaparin | None | CRNMB, day of procedure |
| 61M | Abdominal hernia repair | 35 mg/week | Yes | Enoxaparin | ASA interrupted | CRNMB, day 17 |
| 89M | Foot/hand surgery | 35 mg/week | No | None | None | CRNMB, day 13 |
| 63M | AV fistula repair | 81 mg/week | Yes | Enoxaparin | ASA | CRNMB, day 15 |
| 54M | Sleeve gastrectomy | 37.5 mg/week | Yes | Tinzaparin | None | CRNMB, day 2 |
| 69M | Hemicolectomy | 37.5 mg/week | Yes | Tinzaparin | None | CRNMB, day 14 |
| 57M | Left TKA | 43 mg/week | Yes | Enoxaparin | ASA | CRNMB, day 19 |
| 58M | Nephrolithotomy | 32 mg/week | Yes | Enoxaparin | ASA | CRNMB, day 4 |
| 76M | Cardiac catheterization | 49 mg/week | Yes | Enoxaparin | None | CRNMB, day of procedure |
| 75M | Sural nerve biopsy | 37.5 mg/week | Yes | Enoxaparin | ASA | CRNMB, day 0 + day 2 |
| 81M | Cutaneous/lymph node biopsies | 23.75 mg/week | Yes | Dalteparin | None | CRNMB, day 7 |
| 68M | Back lesion excision | 40 mg/week | Yes | Enoxaparin | ASA | CRNMB, day 10 |
| 75M | Carpal tunnel release | 37.5 mg/week | Yes | Enoxaparin | None | CRNMB, hospitalization |
| 71M | TURP/bladder tumor resection | 17.5 mg/week | Yes | Enoxaparin | None | CRNMB, day 5 |
| 51F | Left TKA | 35 mg/week | Yes | Tinzaparin | None | CRNMB, day 12 |

**Table S5. Adverse events & practice patterns**
